# Promoting and supporting credibility in neuroscience

**DOI:** 10.1177/2398212819844167

**Published:** 2019-04-10

**Authors:** Guillaume A. Rousselet, Georgina Hazell, Anne Cooke, Jeffrey W. Dalley

**Affiliations:** 1 Institute of Neuroscience and Psychology, College of Medical, Veterinary and Life Sciences, University of Glasgow, Glasgow, UK; 2 British Neuroscience Association, Bristol, UK; 3 Department of Psychology, University of Cambridge, Cambridge, UK

## Introduction

A core objective of the British Neuroscience Association (BNA) is to promote and support credibility in neuroscience. Creeping changes in the research culture have created a major problem for science today. Historically, scientific data that were dramatic, novel and positive had been valued and rewarded much more highly than incremental, reproduced or null results. Although novel and positive results are indeed to be celebrated, doing so at the cost of ignoring replication studies or null findings has led to a marked reduction in reproducible, replicable and reliable science research (Fanelli, 2010, 2012).

While the issue of scientific credibility is now being addressed by many research councils, institutes and journals, which support and adopt credibility initiatives, the archaic ‘publish or perish’ attitude still resonates throughout our neuroscience community. Neuroscience can learn much from fields that have already turned the credibility spotlight on themselves (e.g. Psychology), as well as organisations such as the *Centre for Open Science* (COS, USA) and the UK Reproducibility Network (UKRN) who seek to increase the ‘openness, integrity, and reproducibility of scientific research’.

Over the coming years, a core objective of the BNA is to promote and support credibility in neuroscience, facilitating a cultural shift away from ‘publish or perish’ towards one which is best for neuroscience, neuroscientists, policymakers and the public. Among many of our credibility activities, we will lead by example by ensuring that our journal, *Brain and Neuroscience Advances*, exemplifies scientific practices that aim to improve the reproducibility, replicability and reliability of neuroscience research. To support these practices, we are implementing some of the Transparency and Openness Promotion (TOP) guidelines, including badges for open data, open materials and preregistered studies. The journal also offers the Registered Report (RR) article format. In this editorial, we describe our expectations for articles submitted to *Brain and Neuroscience Advances*.

## Reproducibility, replicability and reliability

Three fundamental markers of credibility are the reproducibility, replicability and reliability of neuroscience research. We mainly refer to reproducibility and replicability in this editorial – but to aid understanding it is important to first describe these three terms. As the terms reproducibility and replicability are often used interchangeably, it is useful to define them separately. An analysis can be defined as reproducible if an independent researcher can obtain the same numerical results when provided with data and code from the original study (Peng, 2015). An effect is defined as replicable if a new experiment, following the exact protocol that led to the original result, produces results similar to the original ones. Replicability thus depends in part on the reproducibility of the methods and is also less clearly defined than reproducibility because it depends on defining an acceptable level of similarity for two results. Finally, reliability mainly relates to the accuracy of the scientific tools employed.

## Data and code sharing

The cornerstone of reproducibility is the availability of data and analysis code. While we are not making data sharing compulsory, we request that every article contains a *data sharing statement*, indicating where the data and analysis code can be downloaded. If data are not available, a reason for not sharing must be provided. Sharing on demand by contacting the authors is not a viable option in the short or the long term and will not be accepted as a valid statement (Houtkoop et al., 2018). Articles providing a URL or DOI to a third-party public repository containing their data and analysis code will be flagged by an ‘Open data’ badge.

## Transparent reporting

Transparent reporting is key to replicability, starting with a clear description of sample sizes involved at every level of analysis (Weissgerber et al., 2016). For instance, the number of subjects and the number of measurements per subject should be justified and described in the Methods section. Sample sizes should also be clearly indicated in figures or figure captions and for each analysis, unless sample size is constant across all analyses. Articles providing a URL or DOI to a third-party public repository containing their experimental materials will be flagged by an ‘Open material’ badge. Materials are field dependent and could include, for instance, auditory stimuli and the code to present them to participants, or a detailed lab notebook describing all the steps carried out at the bench.

To let readers assess the results, as much as possible we request detailed illustrations of the observations, irrespective of the outcome of statistical analyses. In particular, we do not accept bar and line charts that hide distributions of observations and valuable information about the nature of the effects (Rousselet et al., 2016; Weissgerber et al., 2015). We expect authors to take advantage of modern software to make the most of their data and convey an informative and nuanced description of the results to the readers (Rousselet et al., 2017; Wickham, 2016). Enough information must also be provided about the statistical tests performed (Weissgerber et al., 2018).

## Transparent contribution reporting

They are many ways authors could have contributed to an article. To recognise and acknowledge this diversity, a *Contributions* section must list the specific roles of everyone involved. To help reporting this important information, we recommend the CRediT taxonomy (https://www.casrai.org/credit.html)

## Statistical reporting

### Graphs

The first step in reporting statistical analyses is to describe the results in detail using graphical representations. In many situations, detailed graphs are sufficient to characterise a dataset without also presenting statistical tests, especially if the goal of a study is to estimate the size of an effect. Along with others, we believe that a focus on estimation is the most productive way to conduct and report statistical analyses (Cumming, 2014; Kruschke and Liddell, 2018).

### Analysis

Whatever the graph choices, authors must justify them explicitly, as well as the choice of statistical tests, alpha level for error control, sample size and hypotheses tested (Lakens et al., 2018). Common choices include using t-tests on means, alpha = 0.05 and a null hypothesis, but these choices are often inappropriate. In particular, many types of variables quantified in neuroscience projects violate the core assumptions of techniques such as standard t-tests, analyses of variance (ANOVAs), correlations and regressions, potentially leading to lower statistical power or increased false positives; robust statistics can address these issues and help provide a better understanding of the data (Wilcox and Rousselet, 2018). Relying on standard techniques can also lead to inaccurate sample size estimation when planning for statistical power. Sample size does not need to be justified based on statistical power: another approach is to focus on estimation accuracy (Peters and Crutzen, 2017; Rothman and Greenland, 2018).

### Significance

Adding the qualifier ‘significant’ or ‘not significant’ after a p value does not add any information. It only provides a false sense of certainty that no statistical technique can provide (Gelman, 2018). Instead, readers should be provided with sufficient information to decide for themselves what they think of the results. Indeed, the American Statistical Association’s (ASA) statement on p values clearly state that ‘Scientific conclusions and business or policy decisions should not be based only on whether a p-value passes a specific threshold’ (Wasserstein and Lazar, 2016). In addition to p values, authors should consider carefully the information provided by effect sizes, confidence intervals and other sources of information, and put that information in context (Amrhein et al., 2018; McShane et al., 2017).

Going beyond p values, assessing the practical significance of research findings is critical, in part because it is trivial to achieve ‘significance’ from noisy measurements or large collections of samples, even in the absence of underlying effects (Loken and Gelman, 2017). The problem gets worse with many implicit or explicit researchers’ degrees of freedom (Forstmeier et al., 2016; Simmons et al., 2011). And when dealing with small samples, filtering by significance leads to inflated effect sizes, or even effect sizes in the wrong direction – the so-called type M and type S errors (Gelman and Carlin, 2014). A more productive approach is to describe the methods and results in as much detail as possible, share data and code and let readers make their own mind about the results, without forcing artificial dichotomies on the readers. In particular, we request that authors declare if all measures and statistical analyses have been reported.

## Registered reports

The introduction of the RR format aims to improve the reproducibility and replicability of neuroscience research. A thorough description of RR is available on the Open Science Framework (OSF) website (https://cos.io/rr/), in particular this FAQ (https://osf.io/gha9f/). In short, unlike standard research articles, RRs have been designed to minimise questionable research practices (such as p-hacking and HARKing) as well as the publishing incentives that promote them (Chambers et al., 2015). The RR format is applicable to standard studies, replication studies and studies planning the analysis of existing datasets. At the core of RR is an innovative reviewing process, in which the Introduction and Methods sections are reviewed before the research is carried out. Articles are thus evaluated solely based on the importance of the topic and the quality of the research methods and analyses, not based on the results. Thus, RRs offer a great way to improve experimental methods to make the most out of lab resources by getting feedback from experts when it matters most: before data collection, not after. Receiving feedback from the scientific community prior to commencing the actual experiments helps improve experimental designs, the choice of tools, data quantification methods and statistical tests. We all make mistakes or are unaware of better alternatives, or both, and this should not be demonised – instead we should work together to find solutions, make the best of our limited resources, which in turn will increase the reproducibility, replicability and reliability of our studies.

Proposals of sufficient quality are approved to progress to the data collection stage. Providing the authors followed the methods discussed and agreed during stage 1 and reached sensible conclusions about the results, the article is accepted for publication no matter how the results turned up.

This two-step process clearly delineates exploratory from confirmatory research (Forstmeier et al., 2016; Wagenmakers et al., 2012), such that readers can trust a study does not suffer from p-hacking and HARKing for instance (Kerr, 1998; Simmons et al., 2011). RRs incorporate other critical features aimed at boosting research credibility, for instance, mandatory data and code sharing for reproducibility, and the demand for at least 90% power to improve replicability.

Why 0.9 power and not the more traditional 0.8? Both are completely arbitrary values. But let us look at this choice from the perspective of replicability: ‘Studies are often designed or claimed to have 80% power against a key alternative when using a 0.05 significance level, although in execution often have less power due to unanticipated problems such as low subject recruitment. Thus, if the alternative is correct and the actual power of two studies is 80%, the chance that the studies will both show P ⩽ 0.05 will at best be only 0.80(0.80) = 64%; furthermore, the chance that one study shows P ⩽ 0.05 and the other does not (and thus will be misinterpreted as showing conflicting results) is 2(0.80)0.20 = 32% or about 1 chance in 3’ (Greenland et al., 2016). With 90% power, the chance that the two studies will show p ⩽ 0.05 will at most be 0.90(0.90) = 81%. This is much better than 64%, although it still leaves the door open for a large amount of apparent discrepancies among studies if the outcomes are judged solely on the basis of p values. Also, power estimation typically assumes that the data do not violate tests’ expectations and that there are no measurement noise and other sources of variability beyond random sampling. As such, the actual power of a line of research will necessarily be lower than anticipated. Hence, we feel that aiming for at least 90% power is entirely justified, given that in practice power will tend to be lower.

## Preregistration

In addition to RR, the journal welcomes the submission of preregistered work, for instance, using the OSF. Unlike RR, preregistered studies are not reviewed before data collection or data analysis. Preregistration affords only some of the benefits of RR: most notably it allows a clear demarcation between confirmatory and exploratory analyses; it also enhances the discoverability of research that might not be ultimately published. If authors can provide a public time-stamped document describing their experimental design and analysis protocol, dated before the start of data collection or data examination, their articles could be awarded a ‘Preregistered’ badge. A badge can also be obtained when only the analyses are preregistered.

## Exploratory research

By promoting RR and confirmatory research, we do not imply that exploratory research should be discouraged. High-quality exploratory research is necessary to the research enterprise by providing useful results that can be used to build theories and generate hypotheses, which in turn can be tested using a confirmatory approach (McIntosh, 2017). After all, some of the best work in neuroscience was exploratory, for instance, the Nobel Prize work of Hubel and Wiesel: ‘Looking back, Hubel considered that their research “was by and large a huge fishing trip”’ (Martin, 2014).

More suboptimal is to present exploratory research as confirmatory, because while it is easy to obtain ‘significant’ results and to write post hoc stories about them, such findings do not tend to replicate and thus undermine the credibility of research. Adding p values to exploratory findings cannot make them more than what they are, certainly not turn them into confirmatory results. In fact, p values can be difficult to interpret for exploratory research (Kruschke and Liddell, 2018; Wagenmakers, 2007).

While we currently do not offer an exploratory report format, we welcome submission of high-quality exploratory work, on its own, or as part of a stage 1 RR submission. Exploratory research should be presented without p values or confidence intervals.

## A bright future for neuroscience

By creating a format that encourages transparent reporting (including negative results), rigorous statistical analyses, sharing at all stages of discovery and highlighting individual authors’ contributions, we hope to increase the reproducibility, replicability and reliability of the research published in *Brain and Neuroscience Advances* and provide benefits to all involved. We also encourage other neuroscience (and non-neuroscience) journals to continue to adopt these initiatives. It is only when we work together as a neuroscience community that we will achieve more productive and beneficial investigations – and in turn improve the trust of the public in our research.
